# Physiotherapist and nurse perspectives on the acceptability and timing of patient-reported outcome measures in clinical practice: Balancing standardisation and flexibility

**DOI:** 10.1007/s11136-026-04277-x

**Published:** 2026-06-06

**Authors:** Jessica Nikolovski, Marika Franklin, Rachael L. Morton, Matilda Armstrong, Gill Hartas, Brad Rossiter, Margaret Fagan, Melissa Tinsley, Jean-Frederic Levesque, Kim Sutherland, Rebecca Mercieca-Bebber, Claire Snyder, Olalekan Lee Aiyegbusi, Rhonda Power, Thelma De Lisser-Howarth, John Paul Troiani, Jane Maher, Joanne Leonard, Katrina Elias, Claudia Rutherford

**Affiliations:** 1 Faculty of Medicine and Health, NHMRC Clinical Trials Centre, University of Sydney, Sydney, Australia; 2 Faculty of Medicine and Health, Susan Wakil School of Nursing and Midwifery, Sydney Quality of Life Office (SQOLO), University of Sydney, Sydney, Australia; 3 The Daffodil Centre, The University of Sydney, a Joint Venture With Cancer Council NSW, Sydney, Australia; 4 Agency for Clinical Innovation, Sydney, Australia; 5 Centre for Primary Health Care and Equity, University of New South Wales, Sydney, Australia; 6 Office for Health and Medical Research, NSW Ministry of Health, Sydney, Australia; 7 Departments of Medicine, Oncology, and Health Policy and Management, Johns Hopkins University School of Medicine and Bloomberg School of Public Health, Baltimore, MD USA; 8 Centre for Patient Reported Outcomes Research (CPROR), Department of Applied Health Sciences, School of Health Sciences, College of Medicine and Health, University of Birmingham, Birmingham , UK; 9 Healthcare Improvement Unit, Quality, Strategy and Improvement, Central Coast Local Health District, Gosford, NSW Australia; 10 Clinical Governance Unit, Northern Sydney Local Health District, St Leonards, Australia; 11 Clinical Governance Unit, Illawarra Shoalhaven Local Health District, Warrawong, Australia; 12 Clinical Governance Unit, Sydney Local Health District, Camperdown, Australia; 13 Clinical Governance Unit, Murrumbidgee Local Health District, Wagga, Australia; 14 Integrated and Community Health, Western Sydney Local Health District, Blacktown, Australia

**Keywords:** Patient-reported outcome measures, Chronic conditions, Qualitative, Acceptability, Timing

## Abstract

**Purpose:**

Patient-reported outcome measures (PROMs), when used at the point-of-care, provide a mechanism to systematically integrate patients' voices into shared decision-making. We examined clinicians’ perspectives on the acceptability and preferred timing of PROM completion in routine clinical care for respiratory, musculoskeletal, cardiac, kidney and diabetic condition management.

**Methods:**

Semi-structured interviews were conducted over videoconference between October 2024 and May 2025. Participants worked at various New South Wales Health clinics, providing care for patients with chronic conditions, and were eligible to collect and review PROMs digitally using the Health Outcomes Patient Experience platform (NSW PRMs-HOPE program). Reflexive thematic analysis was undertaken.

**Results:**

Twenty-two physiotherapists and nurses were interviewed. Acceptability themes included: (1) purpose of PROMs; (2) broader ethical considerations for PROMs collection and use; (3) practical aspects of PROM administration. Findings highlighted the elusiveness of an “ideal” timing for PROMs. Timing themes included: (1) (mis)alignment in timing of PROM administration; (2) preferences for fixed or customised timing and frequency of administration; (3) temporal fit and workflow alignment.

**Conclusion:**

PROMs were reported as most acceptable when their selection, content, and timing aligned with clinical purpose, scope of practice, and existing workflows. Flexibility in PROM administration was perceived to enhance relevance at the point-of-care and support timely, condition-specific clinical conversations and interventions. However, clinicians also recognised that increased flexibility may reduce the comparability of aggregated PROM data across cohorts, highlighting an inherent acceptability trade-off between meeting individual clinical needs and supporting system-level performance monitoring and benchmarking.

**Supplementary Information:**

The online version contains supplementary material available at 10.1007/s11136-026-04277-x.

## Plain Language Summary

People with long‑term health conditions can fill out health surveys called patient‑reported outcome measures (PROMs). These health surveys help patients share how they are coping day to day and how their health condition or treatment is affecting their quality of life. PROMs are used more in healthcare now, but we do not know enough about how clinicians feel about using them or when they should be given to patients to improve their care.

In this study, we spoke with physiotherapists and nurses who use PROMs. They told us what they think about PROMs and when these should be completed by patients.

Physiotherapists and nurses said PROMs can help them better understand their patients, find changes in their patients' health, and support conversations about care. But they also said they needed extra time to help patients fill in PROMs, some of the questions were unclear for patients, there were language and cultural barriers for some patients, and they did not always know how to use the results.

Overall, physiotherapists and nurses wanted more choice in which PROMs they use and when patients complete them. They felt this would make PROMs more useful for patient care. But, too much choice could make it harder to compare results across clinics and to see how well services are looking after people.

**Supplementary Information:**

The online version contains supplementary material available at 10.1007/s11136-026-04277-x.

## Introduction

Patient-reported outcome measures (PROMs) are generic quality of life or condition-specific questionnaires completed by patients that assess symptoms, functional status, and health-related quality of life [1, 2]. There is substantial international literature on PROMs in clinical practice, including evidence on barriers (workflow burden, interpretability) [3, 4], facilitators (integration with multidisciplinary care, electronic capture to improve accessibility and real-time monitoring), and implementation outcomes (patient-clinician communication and symptom detection) [5, 6].

PROMs use in Australian health services has increased over the past decade as an enabler of value-based care [7, 8]. PROMs are now embedded in several state-level programs, which facilitate electronic PROM collection and use across multiple high-burden chronic conditions. Chronic conditions (e.g., osteoporosis/arthritis, heart failure, chronic obstructive pulmonary disease, diabetes, kidney failure) can involve long trajectories, multidisciplinary care, and ongoing symptom fluctuation, making PROMs potentially useful for monitoring, goal-setting, and shared decision-making during clinical encounters [9]. However, real-world adoption varies, and the timing of PROM administration is often inconsistently specified (e.g., “follow-up” or “post-treatment”), thereby limiting clinical actionability [unpublished].

Whether PROMs are used meaningfully in clinical care depends not only on their availability but also on how clinicians experience their fit within their practice. Decisions about if, when, and how PROMs are used are shaped by their clinical purpose, perceived usefulness, compatibility with workflows, patient needs, and service expectations [8]. Understanding these perspectives is critical to explaining why PROMs are enthusiastically adopted in some contexts yet remain peripheral or burdensome in others.

Recognising the growing use of PROMs in managing chronic conditions in clinical practice, we aimed to understand clinician perspectives on the (1) acceptability of PROMs and (2) optimal timing of PROM administration.

## Materials and methods

This study was conducted and reported according to the COnsolidated criteria for REporting Qualitative research checklist (Supplementary File 1) [10].

### Setting

We followed a published protocol that leveraged the existing infrastructure of New South Wales Health, established as part of PROM implementation in New South Wales, Australia (herein, NSW PRMs-HOPE program) [11]. See Box 1 and Table 1 for detailed program information.

**Table 1:** Clinical cohorts of interest and patient-reported outcome measures (PROMs) recommended

Cohort	PROMs recommended
Osteoarthritis chronic care program	PROMIS-29; HOOS/OHS; KOOS/OKS
Osteoporotic refracture prevention	PROMIS-29, FES-1
Diabetes high risk foot services	PROMIS-29, CWIS
Inpatient management of diabetes mellitus	PAID or DDS
Management of chronic obstructive pulmonary disease	PROMIS-29, CAT
Management of chronic heart failure	PROMIS-29, KCCQ-12
Renal supportive care	EQ-5D-5L, IPOS Renal

Box 1. PROM-PATH and NSW PRMs-HOPE This qualitative study is part of a larger research program called **PROM P**resentation, **A**cceptability, **T**iming and **H**ealth service use in New South Wales (PROM-PATH).

The NSW PRMs-HOPE program supports the standardised collection, reporting, and use of patient-reported outcome measures (PROMs and patient-reported experience measures (PREMs)) to inform clinical care, service improvement, and system-level decision-making. Since 2021, the program has collected over 240,000 PRMs from more than 75,000 patients, is embedded within electronic medical records across 1,674 active locations and offers PROMs in 11 languages. PROM data is available for all New South Wales Health staff as 'Read-only' within the electronic medical record, allowing other teams to read and use it clinically.

This study targets New South Wales public clinics participating in the NSW PRMs-HOPE program across seven high-burden chronic conditions to capture cross-specialty variation in PROM use and timing requirements. Clinics outside these cohorts were excluded to maintain governance feasibility.

Table 1 summarises the endorsed PROMs available to participating clinics and their recommended timepoints for completion to contextualise participants’ comments on generic quality of life and condition-specific PROMs and their perceived clinical relevance.

This table reflects endorsed PROMs in the NSW PRMs-HOPE program; however, clinicians using NSW PRMs-HOPE program can access, assign, and use other PROMs available in the platform as they see clinically relevant for their patient(s). All cohorts are recommended to administer PROMs at baseline, every 3 months and then at completion of the program. See Supplementary File 2 for PROM names and copyright information.

### Eligibility criteria

Eligibility criteria are outlined in Box 2.

Box 2. Eligibility criteria Clinicians were eligible for the study if they:

- Understood and spoke English *and*
- Were aged 18 years or over *and*
- Had used the NSW PRMs-HOPE platform to assign or review PROMs *or*
- Were eligible to assign or review PROMs using the NSW PRMs-HOPE platform* and*
- Were a clinician (allied health, medical specialist, nursing professional) involved in the care of patients enrolled in one or more of the clinical cohorts of interest *and*
- Able to complete electronic consent.

Clinics of interest:

- Management of Osteoarthritis (OACCP)
- Osteoporotic Re-fracture Prevention (ORP)
- Chronic Heart Failure (CHF)
- Chronic Obstructive Pulmonary Disease (COPD)
- Renal Supportive Care (End Stage Kidney Disease) (RSC)
- Diabetes High Risk Foot Services (HRFS)
- Inpatient Management of Diabetes Mellitus (IDM)
- Transitional Aged Care Program (TACP), if the clinician sees patients from the above clinics

Clinicians from various specialties, clinical experience (years), and perspectives on PROMs were sought to capture diverse perspectives.

### Recruitment and consent

Ethics approval was granted by the New South Wales Local Health District Human Research Ethics Committee (Study Protocol #X24-0138). Site-specific governance authorisation was obtained for participating health districts.

All health districts in New South Wales (N = 18) were invited to participate in this study; seven agreed. A purposive and convenience recruitment strategy was used (October 2024-May 2025). Patient Reported Measures (PRM) leads (responsible for PRM implementation across their district) emailed a pool of about 70 eligible clinicians an invitation and electronic consent form. JN liaised with PRM leads, responded to queries and contacted consenting clinicians to schedule interviews. JN had no prior relationships with participants.

Reasons for nonparticipation by medical staff and other allied health professionals were collected opportunistically via email replies to invitations and brief follow-up communications from PRM leads. Reasons included: lack of time/interest; clinic not yet onboarded; or PROM administration delegated to nurses. These were not treated as formal data, but we report them to contextualise sample composition.

### Data collection

An interview topic guide (Supplementary File 3) was developed iteratively based on a literature search on seminal papers in the PROMs literature [9, 12] and discussions within a multidisciplinary team. It was piloted with consumer advisors before interviews commenced. Iterations focused on refining question content and sequencing, informed by early interviews and team reflection, to ensure alignment with the study aims. For example, existing evidence reported the acceptability of one quality of life PROM [3]. Thus, we included questions about the acceptability of content in condition-specific and generic quality-of-life PROMs.

A single semi-structured interview with each participant was conducted by JN or MFr, audio-recorded and transcribed verbatim. We did not report data saturation because, in line with Braun and Clarke’s reflexive thematic analysis, it is not considered an appropriate marker of analytic quality; instead, the focus is on generating a rich, coherent, and reflexively developed analysis aligned with the study aims [13].

### Data analysis

Analysis followed a reflexive thematic approach, with themes generated through iterative engagement with the data [14]. Sekhon et al.’s Theoretical Framework of Acceptability was used as a sensitising conceptual lens to inform interpretation of participants’ accounts, particularly relating to how they felt about PROMs (affective attitude), the perceived effort involved (burden), ethical concerns, and perceptions of usefulness and feasibility of PROMs in practice [15]. Broader implementation factors such as workflow integration, digital infrastructure, and service resourcing were interpreted as contextual factors shaping clinicians’ judgements regarding the acceptability of PROMs. Data analysis occurred in parallel with data collection.

Transcripts were entered into QSR NVivo 14 to facilitate data management and coding. JN developed the initial coding tree, which was iteratively refined based on interview data and multidisciplinary team discussions (including project partners, consumer advisors, PRM Program Managers, and research team investigators, including psychologists and sociologists). To construct themes, JN brought together codes to create categories, which then informed sub-themes and, finally, themes.

JN (a female early-career, non-clinical researcher with consumer and youth advocacy experience) practised reflexivity through regular debriefs with the study team to triangulate findings and challenge her assumptions [16–19]. Reflexive discussions focused on how the Theoretical Framework of Acceptability components shaped code construction and theme interpretation (e.g., distinguishing burden from opportunity costs), and on positionality.

## Results

**Fig. 1:**
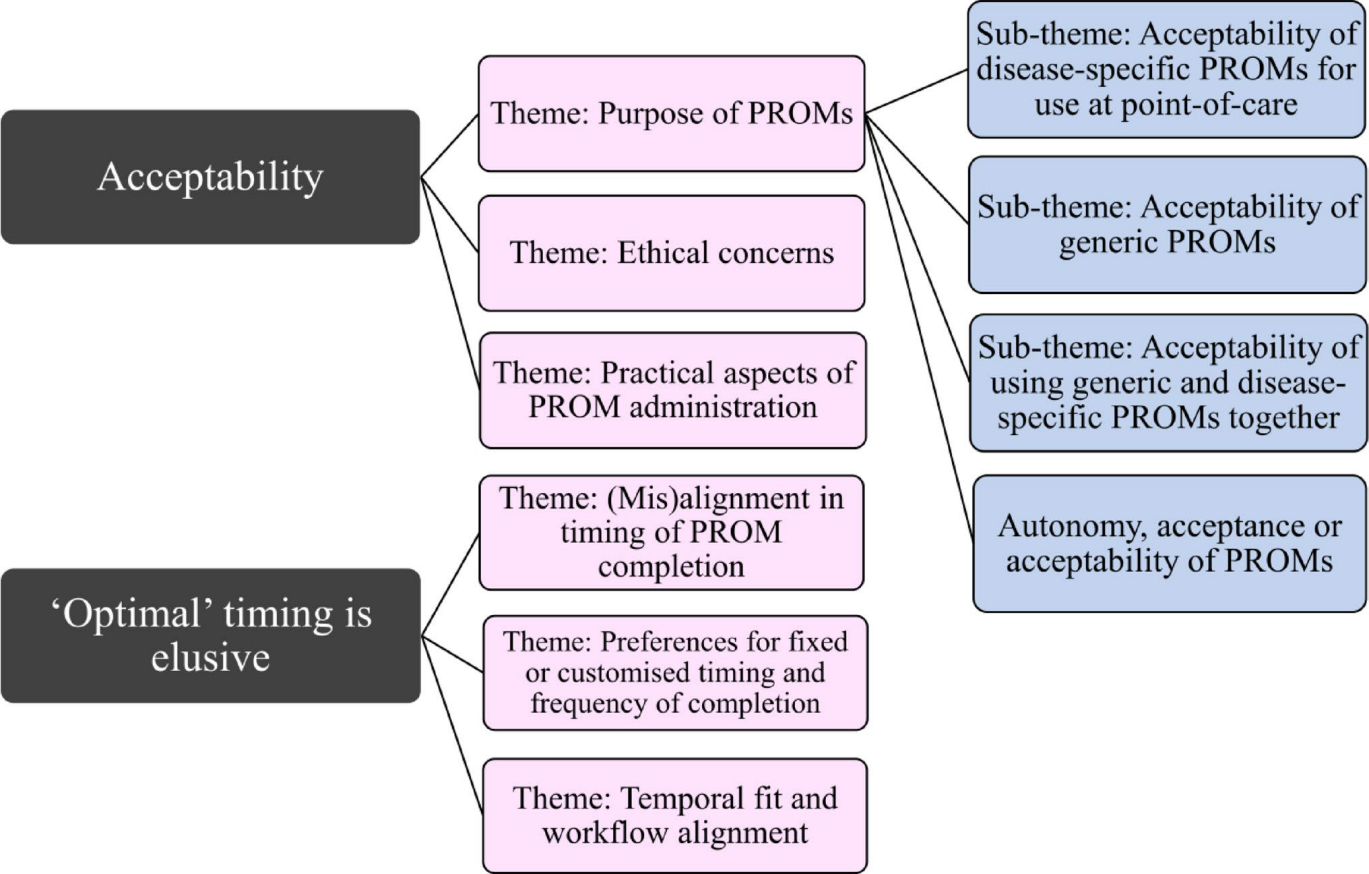
Thematic structure of findings showing themes and sub-themes related to acceptability and timing of PROMs from clinician interviews

Interviews were conducted with 22 participants (17 female; 14 physiotherapists, 8 nurses; Table 2). Interviews ranged from 35 to 71 min (mean = 36 min). Findings are presented within two central topics: Acceptability and Timing (Fig. 1, illustrative quotes Box 3). Themes and sub-themes were mapped to the Theoretical Framework of Acceptability (Table 3). No overall differences in acceptability and timing preferences were identified between nurses and physiotherapists.

**Table 2:** Participant characteristics (n = 22)

Characteristic	n (%)
Local Health District	
Central Coast	2 (9)
Illawarra Shoalhaven	3 (13)
Murrumbidgee	3 (13)
Northern Sydney	1 (4)
Sydney	6 (26)
Western-Sydney	6 (26)
South-East Sydney	1 (4)
Clinical Cohort	
Chronic Heart Failure (CHF)	2 (9)
Chronic Obstructive Pulmonary Disease (COPD)	6 (26)
Inpatient Management of Diabetes Mellitus (IDM)	3 (13)
Renal Supportive Care (RSC)	1 (4)
Transitional Aged Care Program (TACP)	1 (4)
Osteoarthritis Chronic Care Program (OACCP) or Osteoporosis Refracture Program	8 (36)
TACP and OACCP	1 (4)
Profession type	
Nursing (Educator, Consultant, Specialist)	8 (36)
Physiotherapist	8 (36)
Physiotherapist/Program Co-ordinator	6 (26)
Sex	
Females	17 (74)
Males	5 (26)
Continent of Birth	
Australia	16 (73)
Europe	2 (9)
Asia	4 (18)

**Table 3:** Themes and sub-themes mapped to the Theoretical Framework of Acceptability

Component	Affective attitude	Burden	Ethicality	Intervention coherence	Opportunity costs	Perceived effectiveness	Self-efficacy
Definition	How clinicians feel about PROMs	Perceived amount of effort required to participate in PROMs	Extent to which PROMs fit within clinicians’ value system	Extent to which clinicians understand PROMs and how they work	Extent to which benefits or values must be given up to participate in PROMs	Extent to which PROMs are perceived to achieve purpose	Clinicians’ confidence that they can perform behaviours required to participate in PROMs
Themes or sub-themes relating to component	Theme: Purpose of PROMs Sub-Theme: Autonomy, acceptance or acceptability of PROMs Theme: Preferences for fixed or customised timing and frequency of completion	Theme: Practical aspects of PROM administration Sub-theme: Acceptability of using generic and condition-specific PROMs together Theme: (Mis)alignment in timing of PROM completion Theme: Temporal fit and workflow alignment	Sub-Theme: Autonomy, acceptance or acceptability of PROMs Theme: Ethical concerns	Sub-Theme: Autonomy, acceptance or acceptability of PROMs	Theme: Practical aspects of PROMs administration	Theme: Purpose of PROMs Sub-Theme: Autonomy, acceptance or acceptability of PROMs	Theme: Practical aspects of PROMs administration

## Acceptability

### Theme: Purpose of PROMs

PROMs were more likely perceived as acceptable when their use aligned with individual care goals or service models. Sub-themes explored the perspective that when PROMs were not aligned with participants’ preferences without adequate flexibility, they were more likely to be viewed as administratively burdensome and less clinically useful. Participants valued autonomy in selecting which PROMs to use (generic or condition-specific), based on purpose rather than prescription.

PROMs were used for various purposes at the individual level including: (1) to see whether patients were coping with a new diagnosis; (2) to motivate patients to adhere to treatment; (3) general monitoring; (4) to flag concerns that needed addressing; (5) goal-setting based on what patients identified as important to them; (6) determining if an intervention was improving health outcomes, and; (7) facilitate a deeper understanding of patient behaviour (quote [q] 1).

For physiotherapists who collected PROMs only once before their patient’s surgery, the purpose was to provide a snapshot of a patient’s health status rather than a monitoring tool (q2). This was due to the nature of the service rather than because clinicians did not want to follow up patients’ outcomes.

### Sub-theme: Acceptability of condition-specific PROMs for use at point-of-care

Physiotherapists with experience using PROMs before the implementation of the NSW PRMs-HOPE program considered condition-specific PROMs more acceptable than generic PROMs, reporting their content was directly relevant to condition-specific outcomes and treatment planning. Including generic PROMs focused on quality of life, such as PROMIS-29, was considered less acceptable due to concerns about content redundancy, unclear scoring interpretation, and difficulties integrating the results into multidisciplinary team discussions. For these clinicians, generic PROMs introduced burden, without clear added value, reducing overall acceptability.

Physiotherapists from COPD clinics reported using condition-specific PROMs in multidisciplinary teams for decades. Familiarity with how to interpret and action scores facilitated the continued collection of condition-specific PROMs, as generic PROMs were less familiar to general practitioners or specialist staff (q3). Further, these physiotherapists understood how to detect clinically meaningful changes in scores: *“Each score is out of 100, so it is highly sensitive… a reduction of four points shows you’re having an effect” (Physiotherapist, COPD).*

### Sub-theme: Acceptability of generic PROMs

Some nurses and physiotherapists reinforced the importance of person-centred care and how generic PROMs helped broaden the focus of care they provided (q4). Other clinicians reported generic PROMs (e.g., PROMIS-29) were more useful when aggregated for service-level use in quality assurance and benchmarking: “*the PROMIS-29 does not inform practice for us. It simply provides an objective measure of what we're already doing or know of. It's not the Gold Star that's guiding the way in which we provide our care” (Physiotherapist, Program Co-ordinator, TACP).* Standardising which PROMs and their timing was intended to meet reporting requirements, ensure comparability across sites, or align with funding and policy frameworks (q5).

Some physiotherapists in respiratory clinics believed generic PROMs were not validated or tailored to their population. This led to scepticism about their credibility and, therefore, reduced acceptability (q6). A physiotherapist reflected on the importance of knowing current ‘best practice’ to ensure the generic PROMs used were evidence-based (q7).

### Sub-theme: Acceptability of using generic and condition-specific PROMs together

Some participants acknowledged the complementary value of using both generic and condition-specific PROMs. While condition-specific PROMs offered depth about condition-specific concerns and functional outcomes, generic PROMs were seen as helpful in tracking broader functional or psychosocial domains, such as fatigue or depression, which might otherwise be overlooked in condition-specific PROMs (q8). Issues related to the interpretation of results were described (q11).

Participants were concerned that administering multiple PROMs would burden them and their patients. They preferred a single PROM, ideally balancing condition-specificity with comprehensiveness across psychosocial domains (q9). If the clinic was running behind or a patient was perceived to take too long to complete their PROM(s), one physiotherapist described administering only PROMIS-29, because it was shorter than the condition-specific PROM, not because the content was more acceptable. On an individual patient decision-making level, collecting both generic and condition-specific measures at set time-points did not provide information that directly improved patients’ health outcomes (q10).

### Sub-theme: Autonomy, acceptance or acceptability of PROMs

Some participants described the introduction of generic PROMs as mandated (q12 and 13). These individuals continued to use existing PROMs collected in the clinic, in addition to those specified in Table 1. Speaking to the influence of a systematic and coordinated approach to PROMs collection and use in practice, one physiotherapist stated: *“prior to using the PROMIS-29 we didn't use one at all, so. We are led by the Ministry essentially with a lot of the things that we do. So the Ministry says jump, we say how high?” (Program Co-ordinator, TACP).* A lack of perceived autonomy in deciding whether to opt-in to the NSW PRMs-HOPE program, which PROM(s) to use, and their timing of PROM administration, reduced the acceptability of PROMs overall. These factors resulted in passive acceptance- following the process without fully integrating PROMs into clinical decision-making or seeing them as beneficial to patient care.

### Theme: Broader ethical considerations for PROMs collection and use

Ethical judgements about PROM use encompassed three interrelated but conceptually distinct concerns: informed consent and potential patient distress; availability of translations; cultural and linguistic suitability for patients from diverse cultural and linguistic backgrounds or with limited English proficiency; and workforce preparedness to interpret and respond to PROM data (especially psychosocial domains).

Participants believed PROM collection needed to align with the availability of clinicians with specialised expertise and training in interpreting and acting on PROM data, which was often outside the specialty or training of those tasked with collecting PROMs (q14). Employee support services were offered to this manager's staff to support them when faced with complex topics of mental health and suicidality. Another physiotherapist explained that more junior staff felt uncomfortable addressing some topics, such as mental health.

A few participants believed that patients who enrolled in NSW PRMs-HOPE program did not understand what they were consenting to. They believed that once patients consented to participate in the NSW PRMs-HOPE program, they could not withdraw consent (q15). As service managers felt obliged to have high PROM completion rates, they ensured that every patient completed their assigned PROMs (q16). When asked whether they would collect PROMs if their clinic was not already participating in the NSW PRMs-HOPE program, the aforementioned service manager was unsure whether they would use them.

Some questions were reported to not be culturally appropriate or easily translatable (q17). These factors, combined with low health literacy, were perceived as confusing to patients who were culturally and linguistically diverse. This led to uncertainty about how to interpret PROM data, given differences in how interpreters translated PROM content across languages (q18).

Some reported PROMs may not be acceptable for patients from culturally and linguistically diverse backgrounds due to differences in how these patients perceived health. This may contribute to reporting higher symptom severity or more symptoms, or choosing not to answer questions about socially taboo topics and was, therefore, perceived to not be a good use of time during clinical encounters (q19 and 20). One clinician described having limited access to additional resources to support patients from culturally and linguistically diverse backgrounds, such as online education they used for English-speaking patients. Even though the NSW PRMs-HOPE program is translated end-to-end, they wished for health system-wide access to additional translated resources when issues from PROMs were identified (q21).

Some were concerned that PROMs would cause unnecessary distress for patients who are culturally and linguistically diverse as they reflect on the questions; therefore, PROMs were not used (q22).

### Theme: Practical aspects of PROM collection and use

Participants highlighted a range of practical considerations that influenced acceptability, such as the mode of administration; storage and integration of PROM data within existing electronic medical records; timely and secure access to patient-reported information at the point of care; and the long-term sustainability of the systems used to collect and manage PROMs.

The NSW PRMs-HOPE program allowed PROMs to be administered digitally, on paper, or verbally by clinicians who manually recorded responses into the platform, depending on preference and context. Clinicians in our study reported all methods of completion. For some, electronic PROMs reduced administrative burden and facilitated timely access to results (q23). However, others reported that a large cohort of their patients were older, with poor digital literacy, so a lot of time was spent teaching them how to use the NSW PRMs-HOPE program (q24). In these instances, paper-based PROMs were described as quicker to administer at the point of care, circumventing barriers related to technological competence.

Paper-based completion shifted the administrative burden to nurses, physiotherapists and administrative staff. In the post-completion phase, responses were manually entered into the platform and results interpreted before consultations, reducing efficiency and raising concerns about data-entry errors.

Benefits of having translated PROMs available digitally were described, though not having translations available in a print format was seen as a barrier to accessibility for some patients. One physiotherapist described having access to an iPad for translations during the NSW PRMs-HOPE program pilot phase, but was required to return the iPad once the pilot ended (q26).

## ‘Optimal’ timing of PROMs was elusive

There was no consensus on the ‘optimal’ time-points and frequency of PROM completion across or within clinical cohorts. ‘Optimal’ timing was influenced by clinic workflows, symptom trajectories and the type and timing of care provided within the clinic. A key enabler for collection and use of PROMs at the point of care was flexibility in when and how often PROMs were administered.

### Theme: (Mis)alignment in timing of PROM administration

There was a temporal misalignment between the administration of PROMs and the realities of clinical care delivery. PROMs were often completed before clinic visits or at pre-determined follow-up periods (three-monthly over 12 months) rather than synchronised with treatment changes, or symptom fluctuations: *“So the ideal situation could be to do it when they come into hospitals [for an exacerbation]. But we don’t pick up exacerbations… patients in my population barely have energy to answer the questions” (COPD, Physiotherapist)*. Practicalities and realities of treating severely unwell patients were barriers to participants administering PROMs at their preferred timing for clinical decision making and/or when was most acceptable and appropriate for patients.

There were mixed views about optimal recall periods in PROMs. Some indicated no concerns, whilst others believed that more expansive recall periods (e.g., four weeks for the EQ-5D-5L) might affect recall accuracy (q27).

### Theme: Preferences for fixed or customised timing and frequency of administration

Overall, participants desired more adaptive timing and frequency, where PROMs could be administered at clinically meaningful timepoints (e.g., symptom exacerbation, post-intervention, or when new concerns emerged) (q28). The perceived inability to customise PROM timepoints to align with patient care reduced acceptability. However, it is unclear why they believed they did not have the opportunity to change the timepoints of administration.

Specifically, in OACCP cohorts, physiotherapists providing care within a multidisciplinary team for a 12-month rehabilitation program believed that the most clinically informative time points would be at baseline, three and twelve months into the program, to align with general trajectories of patient improvement. They thought current additional data collection at six and nine months added to workloads without further valuable information to direct care, unless the patient verbally reported having an escalation in symptoms (q29). An interval of three months between baseline and first follow-up PROM was perceived as useful for physiotherapists providing an exercise and education intervention (q30).

### Theme: Temporal fit and workflow alignment

Clinicians emphasised that the timing of PROM administration significantly affected their practical acceptability and perceived usefulness in routine care. Rather than enhancing efficiency, PROMs were often seen as a time-adding task, requiring effort to interpret data, or explain the purpose or results to patients, especially when administered at times that misaligned with clinical relevance or resource availability.

Preferences varied, but many clinicians favoured PROMs that could be completed and reviewed before or during consultations. This approach was considered timesaving and patient-centred, supporting more focused conversations, narrowing down concerns, and therefore supporting shared decision-making. In contrast, PROMs completed outside clinical interactions were often overlooked or outdated for real-time decision-making (q31).

Box 3. Illustrative quotes by themes and sub-themes **ACCEPTABILITY**

**Theme: Purpose of PROMs**

**Quote 1:** “I had a patient who I was getting nowhere with him…[so I gave him PAID]…he came to that question about fear of hypoglycemia and said ‘this is a biggie for me’. It helped me to narrow down the real issue and it helped them to open up” (Nurse, IDM).

**Quote 2:** “I use it as a baseline measure” (Physiotherapist, OACCP).

**Sub-theme:** ***Acceptability of condition-specific PROMs for use at point-of-care***

**Quote 3:** “I have no idea whether the GP actually knows what the PROMIS-29 is. They would have no idea. Like our advanced trainee” (Nurse, COPD).

***Sub-theme: Acceptability of generic PROMs***

**Quote 4:** “Levels of fatigue, energy, participation in social settings, sleep and mental well-being. I think it's a good way of getting a sense of how people are doing more holistically, not just specifically about their heart health” (Nurse, CHF).

**Quote 5:** “I look at pooled data to effectively judge if the programme is working” (Physiotherapist, OACCP).

**Quote 6:** “We are using PROMIS-29, but it's not really validated for respiratory patients, but for some reason we ended up using it. I don’t really know why” (Physiotherapist, COPD).

**Quote 7:** “Evidence-based practise is always changing. So yes, we use the PROMIS-29 now, but I'm very mindful that it takes 10 years for evidence-based practice to get implemented. Why are we waiting 10 years?” (Physiotherapist, Program Co-ordinator, CHF).

***Sub-theme: Acceptability of using generic and condition-specific PROMs together***

**Quote 8:** “I guess as physios we’re pretty good at the musculoskeletal side of things than we are in the mental health side of things” (Physiotherapist, OACCP).

**Quote 9:** “The PROMIS-29 is fairly straightforward and quite short, whereas the HOOS and KOOS are more lengthy… a lot of [the questions] are all the same sort of thing” (Physiotherapist, OACCP).

**Quote 10:** “I think that the belief system, from the clinician perspective, is ‘this is just another piece of paperwork that I've got to do.’ That's not going to significantly change.” (Nurse, COPD).

**Quote 11:** “Sometimes the anxiety and depression component [of PROMIS-29] doesn't match up with HAD. For example, they might score high on the HAD, high anxiety and high depression, but on the PROMIS-29 it just shows mild…then you think then which one do we go off?” (Physiotherapist, COPD).

***Sub-Theme: Autonomy, acceptance or acceptability of PROMs***

**Quote 12:** “PROMIS-29 was imposed on us as the option when the HOPE database came about because we were doing a different survey” (Physiotherapist, Program Co-ordinator, CHF).

**Quote 13:** “We've been asked to do PROMIS-29 [but it] overlaps the questionnaires that we already do as standard now” (Nurse, COPD).

**Theme: Broader ethical considerations for PROMs collection and use**

**Quote 14:** “You're always going back to who is asking this? So allied health assistant, someone that has received a certificate in health, they don't understand everything” (Physiotherapist, Program Co-ordinator, TACP).

**Quote 15:** “Clients consent at the start to do [the PROM]. And then one of the things I've continued to advocate across multiple platforms has been our client cohort removes consent… the problem is it looks like we have incomplete surveys [because] if they remove consent, we can’t do the survey” (Physiotherapist, Program Co-ordinator, TACP).

**Quote 16:** “I think as long as [PROMs] get collected, that's the main thing. I’m just quite tenacious about making sure that I collect everything” (Physiotherapist, OACCP).

**Quote 17:** “Interpreters have made comments that some of the questions are very hard to interpret because there's not a particular word [in the other language]; for example, the first question will be, ‘do you feel hopeless’? And then the next question is ‘do you feel helpless?’” (Nurse, COPD).

**Quote 18:** “It's also got to do with different dialects. That then changes the interpretation of the question, and then that changes the way the patient responds to the questionnaires” (Physiotherapist, COPD, TACP).

**Quote 19:** “What's the cultural belief? That we should rest? We should take medications? Exercise is not part of the cultural beliefs. So I think that's a big part of it, of the over reporting, and definitely anxiety, that's just not well addressed” (Physiotherapist, COPD).

**Quote 20:** “There's definitely a generational culture difference in terms of how healthy is being conceived…you're going to talk to an older Asian person about mental health issues… filling in these questionnaires, I just think it's absolutely not worth the time” (Physiotherapist, COPD, TACP).

**Quote 21:** “It’s harder for me to identify that they've got a problem because there's so, so much less that I can direct them to. For example, there's a mental health programme that's free for clinicians to refer patients into, and they've got an insomnia course, which I'll refer a lot of people into. But it's only in English.” (Physiotherapist, OACCP).

**Quote 22:** “I don't think it's that valuable. If anything, I find if they're going to read these questions, they might actually feel worse before they answer it… having parents myself who don't really speak much English, you have to treat them a different way. So sometimes I don't even use it for that reason.” (Physiotherapist, COPD).

**Theme: Practical aspects of PROMs collection and use**

**Quote 23:** “Before we had to manually score them, but now just entering it on HOPE. It just gives us the number. So that's been really helpful” (Nurse, COPD).

**Quote 24:** “For our cohort of patients, we would still have to read it out anyway to patients because, with the iPad, they found it difficult, the writing a bit too small on the iPad” (Nurse, COPD).

**Quote 25:** “Since our cohort is the older generation, it was very tricky for them to do it on an iPad and some of them may not be tech savvy or had their email. They don’t even know how to use a smartphone.” (Nurse, COPD).

**Quote 26:** Clinicians with resources to sustain electronic PROM collection in clinic explained: “we can then change the language. So that is nice that we can do that. It would be nice if we could access all of those [PROMs] in paper format as well” (Physiotherapist, Program Co-ordinator, CHF).

**TIMING**

**Theme: (Mis)alignment in timing of PROM administration**

**Quote 27:** “PROMIS-29 does ask a week anyway. I think patients do find it more difficult to recall what was happening 4 weeks ago compared to one week ago… if it's a month, you're sort of looking at the bigger picture, like an average over a longer period of time. I think either is probably okay” (Physiotherapist, Program Co-ordinator, OACCP).

**Theme: Preferences for fixed or customised timing and frequency of administration**

**Quote 28:** “If they make it mandatory that you have to do it every time I think it takes away from your engagement. I'm hoping that it doesn't become a task that you have to do. The consult won't flow as well” (Nurse, IDM).

**Quote 29:** “I guess if someone’s coming in saying my hip pain is much worse now, the last three months have been hell. I'm feeling worse. Well, yes, I can look at those and see that they've gone down and maybe their walk tests have suffered as well” (Physiotherapist, Program Co-ordinator, OACCP).

**Quote 30:** “Three months is enough time for someone participating in conservative management for us to be able to pick up noticeable changes. We don't have to wait six months [for a PROM] to identify if our treatment is working” (Physiotherapist, OACCP).

**Theme: Temporal fit and workflow alignment**

**Quote 31:** “At times it is very difficult- you can’t find [any support]. By the time you do, the patient might have lost momentum and they’ll just say “I just really like talking to you, I feel so much better” (Physiotherapist, OACCP).

## Discussion

This study highlighted views about acceptability and ‘optimal’ timing of PROMs in chronic condition management were dependent on: perceived purpose (e.g., individual or service level), ethical concerns, type and duration of care, preferences for mode of completion, content coverage, timing relative to disease trajectory and clinical encounters, and workflow alignment. Clinicians faced challenges in balancing the ideal timing and mode for PROM administration with the practical realities of busy clinical workflows, time constraints, and competing priorities, all of which are known barriers to PROM uptake in clinical care settings [20–25]. Extensive literature and practical guidelines exist on the implementation of PROMs in clinical practice to address known barriers at local implementation [26–29]. Our contribution is not to claim novelty of PROMs in clinical practice, but to situate clinician views within cross-specialty state-level implementation and to consider PROM timing as a determinant of clinical utility and acceptability in chronic care.

Clinicians’ views on PROM acceptability ranged. Positive accounts related to perceptions they promoted holistic patient assessment and care. Negative perceptions were reported when PROMs were poorly integrated into workflows, the purpose was unclear, or clinicians felt unprepared to interpret and act on data, especially psychosocial domains. PROM programmes should clarify objectives (individual care, service benchmarking, or both), then align measure selection, resourcing, and training accordingly. National guidance from the Australian Commission on Safety and Quality in Health Care stresses purpose‑driven implementation and stakeholder engagement [30, 31]; the International Society of Quality Of Life research (ISOQOL) emphasises provider capability, opportunity, and motivation to address burden, coherence, and adoption barriers [26, 27].

While a previous study reported high acceptability of a single generic PROM [3], our findings present a more complex picture of PROMs collection. Specifically, we highlight the concurrent use of local, state and national-level initiatives, which have not been described in earlier studies. Clinicians in our study wanted to use additional PROMs outside of the NSW PRMs-HOPE program, which increased workloads and created duplication of PROM content. In general, issues we reported about the content within PROMs, such as duplication of items, length, and complicated or irrelevant questions, are known factors that contribute to reduced acceptability [32]. Preferences for condition-specific PROMs, because of their specificity and sensitivity, reflect a broader tension between assessing domains to direct individual-level decision-making, and the need to promote holistic, patient-centred care through standardised measures that span multiple specialties so data may be aggregated for quality assurance and benchmarking activities. As most clinicians in this study did not use PROMs data for broader service-level purposes, they were reluctant to work with patients to collect PROMs data. To motivate PROM collection and use, frontline staff should be engaged in implementation planning and feedback loops, such as service-level data reports [33].

When PROM implementation is misaligned with existing clinical workflows, it can disrupt care delivery and reduce acceptability. As previously reported, the timing of PROM completion should reflect the trajectory of disease activity and symptoms. Collecting PROMs during periods of stability may not capture meaningful changes or patient concerns, while missing periods of exacerbation or acute symptoms limits the clinical value of the data [3]. The challenge is that clinicians may need to measure PROMs more frequently during flare-ups, whereas from a system-level perspective, health-related quality of life needs to be measured regularly to anticipate exacerbations. Additionally, flare-ups may be times when more people are unable to answer PROMs given the nature of their symptoms (e.g., shortness of breath or dizziness). Adopting fixed intervals of PROM administration (for comparability and quality assurance) with event‑triggered administration of condition‑specific PROMs (for clinical actionability) could be a pragmatic way to balance service and individual uses for PROMs [33]. International initiatives like the International Consortium for Health Outcomes Measurement (ICHOM) support this approach [34].

Evidence suggests electronic PROMs are a time-saving and cost-minimising alternative to paper-based completion [35, 36]. Our study specifically reported this in the recording and scoring PROMs because of immediate access to scores and visualisation of data. Yet, electronic PROMs increased administrative workload when onboarding patients and troubleshooting technical issues. Clinicians noted this challenge may persist for the next few decades, given the age and digital literacy of their patients.

To reduce healthcare inequity, opportunities are needed for all patients to provide feedback on their health to inform care through initiatives like PROMs. A recent systematic review reported a global lack of targeted, culturally specific and appropriate strategies for PROM completion among culturally and linguistically diverse populations, contributing to the underuse of PROMs [37]. Some clinicians reported not asking some patients to complete PROMs if they perceived cognitive decline or cultural or language barriers. While these decisions were often made with good intent, such as avoiding distress, they risked reinforcing inequities by excluding already underserved populations from contributing their perspectives to clinical decision-making. At the meso level, their PROM data cannot be used to guide resource allocation, planning, and support. This suggests a broader systemic issue around gatekeepers in healthcare (i.e., those who control access to resources, services, and knowledge) in creating or removing opportunities for patients to contribute to clinical decision-making [38].

### Strengths and limitations

This study offers several strengths, including in-depth insights into the optimal timing of PROMs administration, addressing a gap in the evidence. Recruitment from a well-established PROM program ensured relevance to clinical practice. The seven participating LHDs included four metropolitan, two regional, and one rural district, meaning that the findings are inclusive of districts serving remote populations and those with different socio-economic and health system characteristics. Findings are context specific to the NSW PRMs-HOPE program. While they may be transferable to similar clinical settings, further research is needed to explore their applicability across different health systems or programmes with differing service models or funding.

There was an underrepresentation of other clinicians (e.g., medical specialists, allied health) despite numerous targeted recruitment rounds. The professional composition of the sample largely reflects the workforce responsible for PROM collection and use within the NSW PRMs-HOPE program, where physiotherapists and clinical nurses are primarily tasked with assigning, reviewing, and acting on PROM data at the point-of-care. Consequently, the findings emphasise operational and workflow-related considerations of PROM utility and timing and may underrepresent perspectives focused on diagnostic or outcome-driven decision making typically held by medical specialists.

## Conclusion

Flexibility in the timing and type of PROMs collected is necessary to enable personalised care that aligns with patient and clinician preferences, clinical contexts, disease trajectories, and intended uses. This, in turn, may increase patient and clinician engagement with and the utility of PROMs. Standardising which PROMs (particularly generic PROMs) and their timing of administration can assist broader service-level quality assurance and benchmarking but can decrease clinician acceptability at the practice level.

## Supplementary Information

Below is the link to the electronic supplementary material.

Supplementary Material 1.

Supplementary Material 2.

Supplementary Material 3.

## Data Availability

De-identified participant quotes are available within this publication.

## Abbreviations

PROM: Patient-reported outcome measure(s)
PROM-PATH: Research program: Patient-Reported Outcome Measures Presentation, Acceptability, Timing and Health service use in New South Wales
NSW PRMs-HOPE program: New South Wales Patient-Reported Measures Health Outcomes and Patient Experience platform
