# Supplementary material for: Physiotherapist and nurse perspectives on the acceptability and timing of patient-reported outcome measures in clinical practice: Balancing standardisation and flexibility
Source: Qual Life Res. 2026 Jun 6;35(7):177. doi: 10.1007/s11136-026-04277-x (PMC13242462; doi:10.1007/s11136-026-04277-x)
Supplement: Supplementary file 1 — Supplementary Material 1. [file 11136_2026_4277_MOESM1_ESM.docx]

**Supplementary File 2. List of patient-reported outcome measures, their abbreviations, and their trademark information**

**CAT**: Chronic Obstructive Pulmonary Disease Assessment Task. COPD Assessment Test and the CAT logo is a trademark of the GlaxoSmithKline group of companies© 2009 GlaxoSmithKline group of companies. All rights reserved. Last Updated: February 24, 2012

**CWIS**: Cardiff Wound Impact Schedule ©WHRU 1997; DDS: Diabetes Distress Scale DS 12.1.17 © Behavioral Diabetes Institute

**EQ-5D-5L**: GroupEuro-QoL 5 dimensions, 5 levels UK (English) © 2009 EuroQol Group EQ-5D™ is a trademark of the EuroQol

**FES-1**: Falls Efficacy Scale – International Copyright under Creative Commons Agreement (via MAPI <https://eprovide.mapi-trust.org/instruments/falls-efficacy-scale-international>)

**HOOS**: Hip disability and Osteoarthritis Outcome Score English version LK1.1; IPOS Renal: Integrated Palliative Outcome Score – Renal P7-EN 21/05/2015

**KCCQ-12**: Kansas City Cardiomyopathy Questionnaire Copyright ã 2012 John Spertus, MD, MPH

**KOOS**: Knee injury and Osteoarthritis Outcome Score English version LK1.0

**OHS**: Oxford Hip Score© Department of Public Health, University of Oxford, Old Road Campus, Oxford OX3 7LF, UK

**OKS**: Oxford Knee Score ©Department of Public Health, University of Oxford, Old Road Campus, Oxford OX3 7LF, UK

**PAID**: Problem Areas in Diabetes © Joslin Diabetes Center, 1999 (www.joslin.org). All rights reserved.

**PROMIS-29**: Patient-Reported Outcomes Measurement Information System 29-item Profile. Participant Format © 2008-2013 PROMIS Health Organization and PROMIS Cooperative Group.
