# Supplementary material for: Physiotherapist and nurse perspectives on the acceptability and timing of patient-reported outcome measures in clinical practice: Balancing standardisation and flexibility
Source: Qual Life Res. 2026 Jun 6;35(7):177. doi: 10.1007/s11136-026-04277-x (PMC13242462; doi:10.1007/s11136-026-04277-x)
Supplement: Supplementary file 2 — Supplementary Material 2. [file 11136_2026_4277_MOESM2_ESM.docx]

### Supplementary File 3. Interview topic guide

Introductory questions:

1. Which [redacted] clinic(s) or community centre(s) are you currently practicing at?
2. How many years have you been practicing for [redacted]?
3. Can you tell me a little bit about your experiences working with people with [INSERT chronic condition/s], and the type and timing of care that you provide?
4. How many years have you been using [redacted] in your practice? What about PROMS?
5. Can you tell me a bit about your experiences of using PROMs?
   1. Probe: are PROMs used with all patients, specific patients, at specific time points?
   2. Probe: how relevant / important do you find PROMs for chronic condition care in general, to interactions with patients, for reflecting on your practice, to implementing person--centred care, resource allocation, those sorts of things.
   3. Probe: who else might use PROMs in your clinic/centre, how and by who? How is this info shared / used within a multidisciplinary team within or across clinics, systems, sectors?
   4. Have you had experience in using PROMs with CALD patients? Which languages/cultures?
6. In terms of your PROMs use which PROMs do you use?
   1. Probe: And how do you decide which PROM to use?
7. How are PROMs administered in your clinic?
8. What information are patients provided about PROMs when you invite them to complete them?
9. From your perspective, how do you think patients use information collected in PROMs?

**MAIN TOPICS**

**Suitability of PROMs:**

1. Can you reflect on why you use PROMs in your clinic?
   1. Probe: convenient, easy to use, accessible, informative, mandatory, for screening, shared decision making, diagnosis, monitoring, to share info with patients or other health professionals, improve practice?
   2. Probe: How relevant are PROMs for [insert chronic condition/s] care in general, to interactions with patients, for reflecting on their own practice, to implementing person-centred care, resource allocation etc.
2. How do PROMs inform your patient care?
   1. Probe: How are PROM surveys used in routine care with patients? How are they used for [insert chronic condition/s] management over time?
3. What are some of the benefits of using PROMs?
4. What are some of the challenges or difficulties about using PROMs?
   1. Probe: what about in administering PROMs or using PROM data?
   2. Probe: what about integrating PROMs into day-to-day practice, accessing training or impact on workload?
   3. Probe: what about for benefits or challenges of using PROMs with people with co or multimorbidity?
5. What factors would facilitate the use of PROMs in your practice?
   1. Thinking specifically about the PROMIS-29, what questions do you find most useful? In what way are these questions useful? Which if any questions are not useful? In what ways are these not useful?
6. Are there any topics/issues/questions not currently captured in the PROMs that you use that you think would be useful for supporting the care you provide patients with [insert chronic condition/s]?
7. How well supported is the use of PROMs in your clinic?
   1. Probe: training, resourcing, staff buy-in, those sorts of things.

**Timing of PROMs (general):**

1. I’d like to know more about the clinical presentation and disease trajectory for your patients with [insert chronic condition/s]. Could you please tell me about it from your experience?
2. How do you decide when to administer PROMs?

From my understanding, PROMIS-29 is recommended to be administered [insert current recommended time point] and disease-specific PROMs [insert other disease-specific PROMs] are administered [insert current recommended time point]. How well does this timing of PROM completion align with the disease trajectory for patients with [insert chronic condition/s]? From your perspective, what else needs to be considered in relation to when PROMs are completed for [insert chronic condition]?

**Working with Culturally and Linguistically Diverse (CALD) patients:**

1. I would like to explore your experience when treating culturally and linguistically diverse (CALD) patients. Have you previously allocated PROMs to these patients?
   1. If **no**: what prevented you from using PROMs in the treatment and management of CALD patients with [insert chronic condition/s]?
   2. If **yes**: could you please describe your experience with using PROMs with your CALD patients?
   3. Probe: experience of using PROMs in other languages?
2. What has been helpful for using PROMs with CALD patients?
3. What about any barriers you have experienced to using PROMs with CALD patients?
   1. Probe: what about your experiences of working with interpreters to implement PROMs?
4. How could PROMs be used to support your clinical encounters with CALD patients?
5. What strategies have you or others in your clinic put in place to enable PROM completion by CALD patients?

Overall, how would you describe your experience of using PROMs in your clinic?

What do you see the future looks like in terms of the use of PROMs in your practice or more broadly?
